# Ex-vivo Resection and Small-Bowel Auto-transplantation for the Treatment of Tumors at the Root of the Mesentery

**Published:** 2014-08-01

**Authors:** S. Nikeghbalian, M. Aliakbarian, K. Kazemi, A. R. Shamsaeefar, S. H. Mehdi, A. Bahreini, S. A. Malek-Hosseini

**Affiliations:** 1 Shiraz Transplant Research Center, Shiraz University of Medical Sciences, Shiraz, Iran; 2 Surgical Oncology Research Center, Imam Reza Hospital, Faculty of Medicine, Mashhad University of Medical Sciences, Mashhad, Iran

**Keywords:** *Ex-vivo* resection, Intestine, small, Auto-transplantation, Mesentery, Neoplasms, Mesentery, Auto-transplantation, Mortality

## Abstract

Background: Tumors involving the root of the mesentery are generally regarded as “unresectable” with conventional surgical techniques. Resection with conventional surgery may end in life-threatening complications in these patients. *Ex-vivo* resection and auto-transplantation avoids excessive bleeding and prevents ischemic related damage to the small intestine and other organs.

Objective: To share our experience of *ex-vivo* resection of the tumors with involvement of small bowel mesentery followed by small bowel auto-transplantation.

Methods: In this study, medical records of all the patients who underwent *ex-vivo* resection and auto-transplantation at our center were retrospectively analyzed.

Results: The most common indication for the procedure in our series was locally advanced pancreatic carcinoma. Our survival rate was 50% with a mean±SD follow-up of 10.1±9.8 (range: 0–26) months. Causes of early in-hospital mortality were multi-organ failure, sepsis, and cerebrovascular accident. Recurrence of disease was noted in one patient while one patient developed hepatic metastasis after 20 months of surgery.

Conclusion: *Ex-vivo* resection of the tumor and auto-transplantation is the surgical treatment of choice for the locally advanced abdominal tumors involving the root of the mesentery.

## INTRODUCTION

The progress in the field of solid organ and multivisceral transplantation opens a window for resection of abdominal tumors involving the root of the mesentery by *ex-vivo* resection and auto-transplantation. Before this, tumors involving the root of the mesentery were supposed to be “unresectable” by conventional surgical techniques without life-threatening complications [1,2]. Conventional methods of surgical resection for tumors involving the root of the mesentery result in resection of large portions of the intestine. As a consequence, patients develop short bowel syndrome and remain dependent on either total parenteral nutrition (TPN ) or need intestinal transplantation for the survival [3]. *Ex-vivo* resection of tumor and auto-transplantation not only prevent the development of short bowel syndrome but also provide better chance of obtaining tumor-free resection margin. The R0 resection is an independent factor for the long-term survival of the patient after surgery [4,5]. *Ex-vivo* resection and auto-transplantation has long been in use for other organs such as heart, kidney and liver [6,7]. 

Therefore, in this study, we aimed to share our experience of *ex-vivo* resection of the tumors with involvement of small-bowel mesentery followed by small-bowel auto-transplantation.

## PATIENTS AND METHODS

Between November 2010 and January 2013, 12 patients underwent *ex-vivo* excision of tumor and auto-transplantation at our center. Data of the patients were retrospectively analyzed from their medical records. All patients were thoroughly examined. Distant metastases were excluded by pre-operative radiological examination and during laparotomy in all but one patient in whom at the end of the procedure a single tiny metastatic lesion was discovered in the left lobe of the liver. Left lobe hepatectomy was done at the same operation to get a disease-free status. In another patient, the mass was found to be completely adhered to the left kidney so the left kidney was sacrificed to obtain tumor free resection margin. Two patients were referred from oncology clinic after receiving gemcitabine-based neo-adjuvant chemotherapy for surgical management of localized advanced carcinoma, but other patients did not receive this treatment. Patients who were discovered to have malignancy or positive lymph nodes on histopathology reports were referred to the Oncology Department for gemcitabine-based adjuvant chemotherapy.

Technical Considerations

Complete pancreatoduodenectomy with resection of the celiac and superior mesenteric artery was done in nine patients; in two patients with smaller tumors Whipple procedure was performed (Table 1). In one patient in whom tumor was located in the body of the pancreas, only distal pancreatectomy was needed to remove the tumor completely. The last technical variation is a novel technique and up to our knowledge has not been mentioned earlier in the literature. Unlike other procedures, the duodenum, pancreatic head and stomach were saved and the liver was perfused with an autologus saphenous interposition graft which was anastomosed to the stump of the celiac artery. 

**Table 1:** Patients’ demographics

No.	Gender/age	Final diagnosis	Transplanted organ	Present condition
1	F/52	Pancreatic carcinoma	Small bowel	Expired
2	F/58	Pancreatic pseudotumor	Small bowel and liver	Alive
3	F/32	Pancreatic carcinoma	Small bowel and liver	Expired
4	M/45	Pancreatic carcinoma	Small bowel	Alive
5	M/47	Pancreatic pseudotumor	Small bowel	Expired
6	F/16	Retroperitoneal rhabdomyosarcoma	Small bowel	Expired
7	F/56	Pancreatic carcinoma	Small bowel	Expired
8	M/46	Pancreatic carcinoma	Small bowel	Alive
9	F/55	Gastrointestinal Stromal Tumor	Small bowel	Alive
10	M/50	Pancreatic carcinoma	Small bowel	Alive
11	M/73	Pancreatic carcinoma	Small bowel	Expired
12	M/33	Pancreatic carcinoma	Small bowel	Alive

The small-bowel resection, right extended hemicolectomy, splenectomy and retroperitoneal lymph node dissection (RPLND) were also performed in all patients; partial or complete gastrectomy was done in 11 patients. All tissue specimens were sent for histopathological examination.

In our series, seven patients required interposition vascular grafts for the reconstruction of hepatic artery or portal vein. Allograft iliac vessels from deceased donors were used in the first two patients while in five other patients, autologus saphenous or external jugular veins were used.

There were various techniques for approaching the common hepatic artery involvement. In the first two cases, the liver was also resected during *ex-vivo* resection; liver and small-bowel were then auto-transplanted after tumor removal by bench surgery. This technique had an increased risk for developing complications of anhepatic phase, so we changed the surgical strategy. In other patients, the arterial inflow of the liver was replaced with a temporary interposition graft from infrarenal aorta to the proper hepatic artery using autologus saphenous vein before resection of the organs. Later, the proximal end of this bypass graft was repositioned and anastomosed to the stump of the resected celiac artery before small-bowel auto-transplantation. 

## RESULTS

Out of 12 patients who underwent *ex-vivo* resection of the tumor and auto-transplantation, males outnumbered to females in a ratio of 2:1. The mean±SD age of patients at the time of surgery was 45.7±14.6 (range: 17–73) years. 

Ten patients had pre-operative diagnosis of locally advanced pancreatic carcinoma, one had gastrointestinal stromal tumor (GIST), and another had retroperitoneal rhabdomyosarcoma (Table 1). Two patients who were operated with pre-operative diagnosis of pancreatic carcinoma and who were mimicking locally advanced carcinoma were found to have pseudotumor on post-resection histopathology reports of the specimens. One of them had tissue reaction due to previous ruptured teratoma while the other had chronic pancreatitis with severe peri-pancreatic fibrosis. In one patient tumor tissue closely adhered to the left kidney; therefore, the left kidney was sacrificed to obtain a tumor-free resection margin. Tumor-free resection margins were obtained in all patients on histological examination of the specimens.

Out of 12 patients, 10 had small bowel auto-transplantation; the remaining two had combined liver and small bowel auto-transplantation. After reperfusion, both liver and small bowel reimplanted grafts regain normal color and perfusion.

The mean±SD total procedure time was 714±162 (range: 540–960) minutes. Total cold ischemia time varied between 60 and 210 (mean±SD: 160±68) min; the total warm ischemia time varied from 25 to 65 (mean±SD: 39±18) min. The mean hospital stay was 9.7 (range: 1–24) days. 

The two patients who received deceased donor vascular grafts were on immunosuppressive drugs. Both developed surgical complications and died of complications—one developed severe sepsis, probably due to over-immunosuppression; the other developed vascular graft thrombosis and small-bowel gangrene. After this event, we stopped using allograft interposition grafts.

The survival rate was 50%. Six of our patients died during a mean±SD follow-up of 10.1±9.8 (range: 0–26) months. Three patients died during the initial hospital stay. Causes of in-hospital deaths were multi-organ failure, severe sepsis and cerebrovascular accident. Three patients died two months, three months, and six months after the initial surgery. Causes of late mortality were thrombosis of the allograft, late portal vein thrombosis and recurrence of disease, respectively. 

Nine patients with malignant disease survived more than six weeks. All of them were referred to oncologist for chemotherapy. They all received gemcitabine-based chemotherapy. One patient developed recurrence of disease within six months and died as mentioned above; one patient of pancreatic carcinoma developed multiple hepatic metastasis 20 months post-surgery. This patient had been referred to the oncologist for the systemic chemotherapy. 

## DISCUSSION

Tumors involving the root of the mesentery are almost always supposed to be “unresectable” by the conventional techniques of surgery for several reasons. In an attempt to get tumor-free resection margins in these patients, either total or subtotal enterectomies have to be performed ending in short gut syndrome. TPN therapy is an option but chronic TPN therapy predisposes these patients to develop life-threatening complications such as line-related sepsis and liver failure [8,9]. 

These tumors are in close proximity to the major abdominal vessels supplying solid organs and hollow viscera. During resection of these retroperitoneal tumors, mesenteric vessels may need to be tied or clamped at least temporarily to control bleeding in the operating field. Of note, intestinal epithelium is very sensitive to warm ischemia and ischemic changes may appear just after an hour of clamping of the mesenteric vessels during warm ischemia [10]. On the other hand, intestinal epithelium can withstand cold ischemia for 6–8 hours as shown by Takeyoshi and his colleagues, in their experimental surgery in a canine model [11]. This observation is the basis of *ex-vivo* resection and auto-transplantation in which the University of Wisconsin solution is used to prolong the cold ischemia time without any detrimental effect on the autologous grafts. 

There are several advantages for *ex-vivo* tumor resection and auto-transplantation. Dissection can be done in bloodless field in a timely manner, and thus with better chances of obtaining tumor-free resection margins. Also reconstruction of vascular pedicles can be done if needed and large enterectomies can be avoided with less risk of developing short gut syndrome. After exenteration of abdominal organs, abdominal cavity and retroperitoneal space become clearly visible. Any residual tumor or suspicious area can then easily be resected out. Furthermore, hemostasis can be achieved more effectively.

To date, only few studies have been published in the medical literature on *ex-vivo* resection of tumors involving the root of the mesentery and auto-transplantation [1,2,12-16]. To the best of our knowledge, this is one of the largest series ever reported in medical literature.

The underlying diseases for which this procedure has been done in these studies were mesenteric fibroma, vascular dysplasia of the root of the mesentery, desmoid tumor, mesenteric carcinoid tumor, mesenteric cavernous hemangioma and some cases of pancreatic carcinoma. In our series, the most common indication for the operation was pancreatic cancer involving the root of the mesentery. Obviously, malignant tumors need lymph node dissection and a more extended resection compared to benign lesions, which may increase the morbidity and mortality rates in patients with impaired immune system due to malignancy. This may explain the relative high rate of mortality in our patients. 

Use of cadaveric grafts to replace arteries or veins made us to use immunosuppressive drugs with all their side-effects. The two patients in whom allogenic vascular grafts were used, developed complications and died. So, we have not used this kind of vascular grafts since then.

In conclusion, with refinement in surgical techniques as well as using neoadjuvant and adjuvant treatments, this procedure may lead to further improvement in the prognosis of malignant tumors involving the root of the mesentery. Furthermore, this approach would become the standard surgical treatment for otherwise unresectable benign lesions of the small bowel mesentery. 

## Notes

**FINANCIAL SUPPORT:** None.
